# Spontaneous Loss of Virulence in Natural Populations of Listeria monocytogenes

**DOI:** 10.1128/IAI.00541-17

**Published:** 2017-10-18

**Authors:** Mylène M. Maury, Viviane Chenal-Francisque, Hélène Bracq-Dieye, Lei Han, Alexandre Leclercq, Guillaume Vales, Alexandra Moura, Edith Gouin, Mariela Scortti, Olivier Disson, José A. Vázquez-Boland, Marc Lecuit

**Affiliations:** a Institut Pasteur, National Reference Centre and WHO Collaborating Centre for Listeria, Paris, France; b Institut Pasteur, Biology of Infection Unit, Paris, France; c Microbial Pathogenesis Unit, Medical School (Biomedical Sciences), University of Edinburgh, Edinburgh, United Kingdom; d Inserm U1117, Paris, France; e Division of Infection and Immunity, The Roslin Institute, University of Edinburgh, Edinburgh, United Kingdom; f Institut Pasteur, Bacteria-Cell Interactions Unit, Paris, France; g Paris Descartes University, Sorbonne Paris Cité, Institut Imagine, Necker-Enfants Malades University Hospital, Division of Infectious Diseases and Tropical Medicine, APHP, Paris, France; University of Illinois at Chicago

**Keywords:** Listeria monocytogenes, virulence, hemolysis, genomics, spontaneous mutations

## Abstract

The pathogenesis of Listeria monocytogenes depends on the ability of this bacterium to escape from the phagosome of the host cells via the action of the pore-forming toxin listeriolysin O (LLO). Expression of the LLO-encoding gene (*hly*) requires the transcriptional activator PrfA, and both *hly* and *prfA* genes are essential for L. monocytogenes virulence. Here, we used the hemolytic activity of LLO as a phenotypic marker to screen for spontaneous virulence-attenuating mutations in L. monocytogenes. Sixty nonhemolytic isolates were identified among a collection of 57,820 confirmed L. monocytogenes strains isolated from a variety of sources (0.1%). In most cases (56/60; 93.3%), the nonhemolytic phenotype resulted from nonsense, missense, or frameshift mutations in *prfA*. Five strains carried *hly* mutations leading to a single amino acid substitution (G299V) or a premature stop codon causing strong virulence attenuation in mice. In one strain, both *hly* and *gshF* (encoding a glutathione synthase required for full PrfA activity) were missing due to genomic rearrangements likely caused by a transposable element. The PrfA/LLO loss-of-function (PrfA^−^/LLO^−^) mutants belonged to phylogenetically diverse clades of L. monocytogenes, and most were identified among nonclinical strains (57/60). Consistent with the rare occurrence of loss-of-virulence mutations, we show that *prfA* and *hly* are under purifying selection. Although occurring at a low frequency, PrfA^−^/LLO^−^ mutational events in L. monocytogenes lead to niche restriction and open an evolutionary path for obligate saprophytism in this facultative intracellular pathogen.

## INTRODUCTION

Listeria monocytogenes is a foodborne pathogen that can cause a severe invasive disease, called listeriosis, in people and animals. As a facultative intracellular bacterium, L. monocytogenes has evolved a range of virulence determinants allowing intracellular survival (1, 2). One key virulence factor is listeriolysin O (LLO), a pore-forming toxin responsible for the characteristic β-hemolytic phenotype of L. monocytogenes that allows the bacterium to escape from the phagosome of host cells and replicate intracellularly (3, 4). LLO is encoded by *hly*, located in Listeria pathogenicity island 1 (LIPI-1) (5). Expression of the genes within this central pathogenicity locus, including *hly*, is under the control of the transcriptional activator PrfA, the master regulator of L. monocytogenes virulence genes (6, 7). The hemolytic activity conferred by LLO is considered a cardinal marker for L. monocytogenes detection and/or identification in clinical and food microbiology. L. monocytogenes is divided into four phylogenetic lineages (8–10), 13 serotypes (11) that can be approximated by PCR serogrouping (12), and more than 100 clonal complexes (CCs, as defined by multilocus sequence typing [MLST]) (13), which are unevenly virulent (14). Weakly or nonhemolytic L. monocytogenes strains have been reported (15–19), but the frequency and phylogenetic diversity of the strains displaying an altered hemolysis phenotype are unknown, as well as their underlying genetic and microbiological features.

This study aimed at (i) estimating the frequency of naturally occurring nonhemolytic L. monocytogenes isolates and their distribution among L. monocytogenes lineages and MLST clonal complexes, (ii) understanding the molecular bases of the nonhemolytic phenotype, and (iii) assessing its impact on virulence. By using phenotypic and genomic approaches, mutagenesis, and *in vivo* assays, we show that mutations leading to loss of hemolytic activity in L. monocytogenes, although rare, affect a wide range of clonal complexes of the major lineages I and II and lead to a decreased virulence.

## RESULTS

### Identification and characterization of nonhemolytic L. monocytogenes strains.

**FIG 1:**
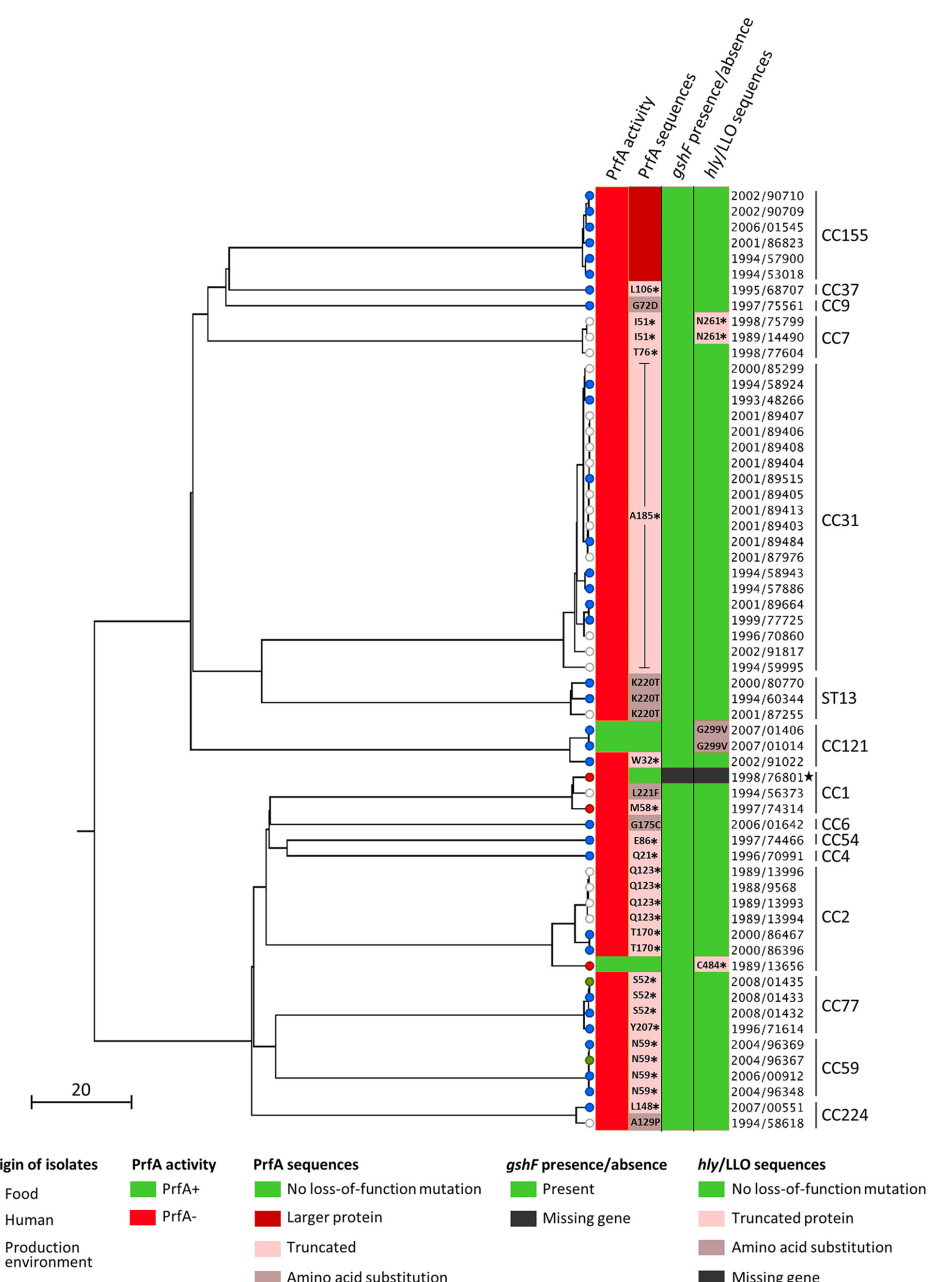
Phylogenetic tree summarizing all the genetic features causing the loss of hemolytic activity among the 60 nonhemolytic L. monocytogenes strains. Single-linkage-based clustering was obtained based on the cgMLST allelic profiles, as described previously (21). The scale bar indicates the percentage of cgMLST similarity. Strain names have been simplified to avoid redundancy and should be preceded by CLIP (Collection of the Institut Pasteur). PrfA activities and mutations (first and second columns, respectively), *gshF* presence/absence profile (third column), and LLO mutations and presence/absence profile (fourth column) are mapped on the phylogeny. The position and the nature of amino acid substitutions are indicated in gray zones. Positions of premature stop codons are indicated next to black asterisks in light pink zones. The absence of *gshF* and *hly* in the CLIP 1998/76801 strain is indicated in black. MLST clonal complexes are shown on the right. The black star highlights the CLIP 1998/76801 strain that contains multiple copies of a transposable element that induced huge genomic rearrangements. ND, not determined (unknown and nonhuman origin).

We examined the prevalence of nonhemolytic L. monocytogenes strains among the 57,820 L. monocytogenes isolates collected between 1987 and 2008 at the French National Reference Centre for Listeria (NRCL) and the WHO Collaborating Centre for Listeria (WHOCCL). Sixty L. monocytogenes isolates (0.1%) were identified as nonhemolytic on horse blood agar plates. These were isolated from food (*n* = 33), food production environments (*n* = 2), nonhuman unknown sources (*n* = 22), and human clinical cases (*n* = 3). Phenotypic characterization using the API Listeria system confirmed all 60 nonhemolytic isolates as L. monocytogenes. These belonged to lineages I (*n* = 23, 38.3%) and II (*n* = 37, 61.7%) and were grouped within serogroups IIa (*n* = 36), IVb (*n* = 13), IIb (*n* = 10), and IIc (*n* = 1) (see Table S1 in the supplemental material). MLST showed that the 60 nonhemolytic isolates belonged to 15 different clonal complexes, including the hypovirulent CC9 (*n* = 1), CC121 (*n* = 3), CC31 (*n* = 20), and sequence type 13 (ST13) (*n* = 3) (14, 20) as well as the hypervirulent CC1 (*n* = 3), CC2 (*n* = 7), CC4 (*n* = 1), and CC6 (*n* = 1) (14) (Fig. 1 and Table S1). Core genome MLST (cgMLST) typing identified 39 different cgMLST types (CTs) (21). Nine CTs comprised more than one strain, suggesting a possible epidemiological link between them (21) (Table S1). In particular, among the 20 nonhemolytic CC31 strains, 10 belonged to CT878, and 2 belonged to CT2659, suggesting that the overrepresentation of CC31 could be in part due to multiple sampling of the same source in the context of an epidemiological investigation. These results show that nonhemolytic strains are phylogenetically very diverse and that the loss of hemolytic activity is caused by independent events across the L. monocytogenes population.

To investigate the impact of the loss of hemolytic activity on L. monocytogenes fitness, we analyzed the growth of all nonhemolytic strains in brain heart infusion (BHI) broth at 22°C and 37°C, using strain EGDe as control (Fig. S1). At 22°C, in a large majority of cases, the growth of nonhemolytic strains was within the same range as that of EGDe, as revealed by the areas under the growth curves (AUCs). In contrast, at 37°C, the temperature at which *prfA* is known to be maximally expressed (22), most of the nonhemolytic strains showed lower growth (lower AUCs) than EGDe. Some of the nonhemolytic strains showed particularly decreased fitness at one or both temperatures: CLIP 2000/86467 (PrfA_T170*_, at 22°C, where the asterisk indicates a truncation at residue T170 of PrfA), CLIP 1998/75799 (PrfA_I51*_-LLO_N261*_, at 37°C), and, at both temperatures, strains CLIP 1998/76801 (Δ*hly*-Δ*gshF*), CLIP 1996/70991 (PrfA_Q21*_), CLIP 1994/58618 (PrfA_A129P_), and CLIP 1996/71614 (PrfA_Y207*_) (Fig. S1).

### Molecular basis of the nonhemolytic phenotype: PrfA variants and activity.

The central regulator of Listeria virulence, PrfA, is required for the expression of a set of key virulence determinants, known as the PrfA regulon, including the *hly* gene (6, 7, 23). Consequently, mutations altering the function of either PrfA or LLO could lead to a nonhemolytic phenotype. Sequence analyses identified frameshifts and missense and nonsense mutations in *prfA* in 56 nonhemolytic strains, leading to amino acid substitutions or protein truncations in PrfA (Fig. 1; Table S1). Phenotypic analysis under PrfA-activating and -nonactivating conditions using the PrfA-dependent virulence factors PlcB (phospholipase C) and Hpt as reporters (see Materials and Methods) (24) confirmed the complete loss of function of the central virulence gene regulator in all of these strains (Fig. 1; Fig. S2).

Forty-three out of the 56 PrfA^−^ strains, distributed in lineages I and II, expressed a truncated PrfA at 14 distinct positions distributed along the entire PrfA protein (Table S1). All analyzed strains of CC59 and CC31 exhibited a truncation at positions 59 and 185, respectively, suggesting a common ancestor for each of these groups of strains. Seven PrfA^−^ strains presented a single amino acid substitution in PrfA compared to the sequence of the reference strain EGDe (GenBank accession number: NC_003210). Among them, one occurred in the β-roll region of PrfA (G72D in strain CLIP 1997/75561, CC9). Mutations located in this region are known to affect PrfA activation or the ability of PrfA to form a stable complex with the RNA polymerase and initiate transcription of the target virulence genes (25–27). One PrfA^−^ mutation occurred in the DNA-binding helix-turn-helix (HTH) domain of PrfA (G175C in strain CLIP 2006/01642, CC6), and two others occurred in its C-terminal part (K220T in strains CLIP 1994/60344, CLIP 2000/80770, and CLIP 2001/87255, all ST13; and L221F in strain CLIP 1994/56373, CC1). These regions are known to be important for the binding of PrfA to PrfA-binding sites of target DNAs (25, 26). In addition, the A129P substitution, located between the β-roll and the hinge αD regions, occurred in a CC224 strain (CLIP 1994/58618). Finally, six of the PrfA^−^ strains, all belonging to CC155, showed a reversion of the *prfA* stop codon due to the insertion of 5 nucleotides at position 712 in the *prfA* sequence, leading to a longer PrfA protein (238 amino acids in EGDe versus 293 amino acids in the CC155 strains of this study).

One of the four nonhemolytic mutants (CC1 strain CLIP 1998/76801) exhibited a wild-type (WT) PrfA sequence compared to that of EGDe but showed a PrfA^−^ phenotype. This observation suggested that a mechanism interfering upstream of PrfA function was affected. Glutathione, synthetized by L. monocytogenes through the glutathione synthase encoded by *gshF* (*lmo2770*), is critical for PrfA activation (28). Interestingly, although it is part of the L. monocytogenes core genome (14, 21), *gshF* was absent in the genome of the CLIP 1998/76801 strain (Fig. 1) (see below), which could explain the absence of PrfA activity in this strain.

### Analysis of spontaneous LLO mutants.

Analysis of *hly* sequences in the 60 nonhemolytic strains identified multiple mutations leading to amino acid substitutions in LLO (Table S1). Several substitutions (N31H, S35L, V438I, and K523S) were identified in at least 48 hemolytic L. monocytogenes strains of our database (∼4,100 genomes), suggesting that they do not cause LLO loss of function. However, an S250N substitution was found only in three nonhemolytic strains of this study (CLIP 2008/01432, 2008/01433, and 2008/01435, all CC77) and could therefore result in LLO loss of function. Since these strains also expressed a truncated and nonactive PrfA, which is sufficient to explain the nonhemolytic phenotype of these strains, we did not pursue this further.

Two out of the three nonhemolytic strains showing a WT PrfA sequence and a PrfA^+^ phenotype (CC121 strains CLIP 2007/01406 and CLIP 2007/01014) exhibited a single amino acid substitution in LLO (the G299V substitution encoded by *hly* [*hly*_G299V_], or LLO_G299V_), which was not present in any of the other strains. The third strain (CC2, CLIP 1989/13656) harbored a premature stop codon at position 484 in LLO (*hly*_C484*_, or LLO_C484*_). The absence of any other specific feature in these three strains that could be linked to the loss of hemolytic activity suggested that the G299V mutation and the truncation at position 484 in LLO could be the cause of the loss of hemolytic activity in these strains. In addition, two CC7 strains expressing a truncated PrfA (CLIP 1998/75799 and CLIP 1989/14490) also showed a premature stop codon in LLO at position 261 (*hly*_N261*_) due to the insertion of one nucleotide.

**FIG 2:**
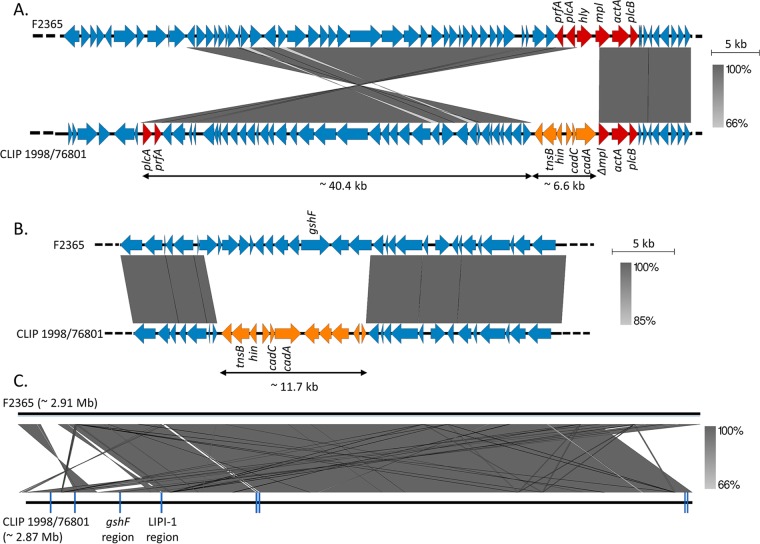
Comparison of the CLIP 1998/76801 and F2365 genomes. (A) Gene content of the LIPI-1 region in F2365 (GenBank accession number NC_002973) in comparison to the corresponding region in the nonhemolytic CLIP 1998/76801 strain, as indicated. LIPI-1 genes are highlighted in red. *mpl* is composed of 1,532 bp in F2365 but 1,133 bp in CLIP 1998/76801. (B) Gene content of the *gshF* region in F2365 in comparison to the corresponding region in CLIP 1998/76801. In panels A and B, genes that are present in CLIP 1998/76801 but absent in F2365 are indicated in orange. Genes encoding the transposition protein (*tnsB*), the DNA invertase (*hin*), and cadmium resistance (*cadA* and *cadC*) are indicated. (C) Global comparison of the F2365 and the CLIP 1998/76801 genomes. Positions of the eight copies of the transposable element are indicated in dark blue. Identity percentages (indicated by gray zones of variable intensities) between sequences were determined by nucleotide BLAST (55). Genome comparisons were performed using Easyfig, version 2.1 (56).

In the CLIP 1998/76801 strain mentioned above, *hly* could not be detected by PCR, and the *hly* region could not be assembled from Illumina reads. In order to resolve this region, we sequenced this strain using single-molecule, real-time (SMRT) sequencing technology (Pacific Biosciences, CA, USA). The CLIP 1998/76801 complete genome (CC1; 2.84 Mb) was compared to the closely related F2365 complete genome (CC1; NCBI accession number NC_002973) as a reference. This showed that the LIPI-1 region had undergone an inversion of more than 40 kb (Fig. 2A). This large rearrangement split LIPI-1 into two parts with a concomitant loss of *hly* and partial truncation of the 5′ region of the adjacent *mpl* gene. Six open reading frames (ORFs) were inserted upstream of *mpl* in CLIP 1998/76801 compared to the sequence of F2365 and comprised genes encoding a transposition protein (*tnsB*) and a DNA invertase (*hin*), which are likely the cause of the rearrangement, as well as cadmium resistance genes (*cadA* and *cadC*) (Fig. 2A).

We confirmed that *gshF* is absent in CLIP 1998/76801, together with 12 other upstream and downstream genes related to sugar metabolism (Fig. 2B). These genes were replaced by 11 ORFs encoding a transposition protein (*tnsB*), a DNA invertase (*hin*), and cadmium resistance (*cadA* and *cadC*), similar to the genes inserted in the LIPI-1 region. In total, eight similar copies of this transposable element were found in the CLIP 1998/76801 genome, as well as many other large rearrangements and deletions (Fig. 2C). Similar transposable elements were detected in one Listeria ivanovii strain in the NCBI database (GenBank accession number KR780025.1; 99% nucleotide identity) and in 128 L. monocytogenes strains (>99.87% nucleotide similarity) of the 4,091 genome sequences available at the NRCL at the time of the study. These strains comprised 14.1% of all the CC1 strains (90/638, representing two distinct monophyletic groups within the phylogeny of CC1 [data not shown]) and all the CC59 strains (*n* = 38). No significant link of this element with food or clinical origins was found within CC1.

### Assessment of *hly* and *prfA* transcription.

In order to test the effect of the identified mutations on *hly* and *prfA* transcription, quantitative reverse transcription-PCRs (qRT-PCRs) were performed for a representative set of nonhemolytic strains (one strain per type of loss-of-hemolysis mutation) (Table S1). All nonhemolytic strains showed *prfA* transcription levels equivalent to or higher than those of EGDe, except for strains CLIP 1998/75799 (PrfA_I51*_-LLO_N261*_ mutations) and CLIP 1998/77604 (PrfA_T76*_ mutation), which showed no amplification, likely due to poor primer annealing (eight mismatches with the *prfA*-R primer) (Fig. S3). As expected, strains with an altered PrfA (amino acid substitution or truncation) showed no or extremely reduced *hly* transcription levels. These results show that for these strains the loss of hemolytic activity is due to *prfA* posttranscriptional events leading to the absence of PrfA activity. In the strain CLIP 2007/01406 (LLO_G299V_), *hly* was transcribed at a level similar to that in EGDe, whereas in CLIP 1989/13656 (LLO_C484*_), *hly* transcription was weaker.

### *In vitro* characterization of the *hly*_G299V_ and *hly*_C484*_ mutations.

In order to characterize the functional impact of the G299V substitution (CLIP 2007/01406 and CLIP 2007/01014) and of the truncation at position 484 in LLO (CLIP 1989/13656), we introduced a plasmid containing either a wild-type *hly* gene (*hly*_WT_) or a mutated version of this gene (*hly*_G299V_ or *hly*_C484*_, encoding LLO_G299V_ and LLO_C484*_, respectively) in an EGDΔ*hly* strain. While EGDΔ*hly*::pPL2-*hly*_WT_ was hemolytic, EGDΔ*hly*::pPL2-*hly*_G299V_ or EGDΔ*hly*::pPL2-*hly*_C484*_ remained nonhemolytic, as assessed on Columbia horse blood agar plates. These results demonstrate that the *hly*_G299V_ and *hly*_C484*_ mutations are responsible for the absence of hemolytic activity in the strains CLIP 2007/01406, CLIP 2007/01014, and CLIP 1989/13656.

**FIG 3:**
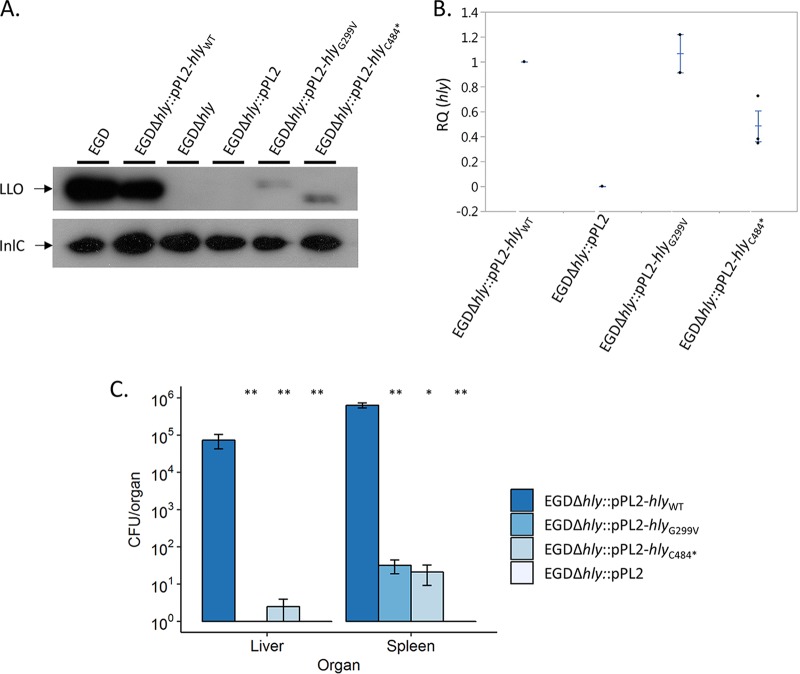
Characterization of the G299V substitution in LLO and the truncated LLO at position 484. (A) Western blotting of the culture supernatants of EGD and EGDΔ*hly* complemented or not with the pPL2 plasmid alone or containing the *hly*_WT_, *hly*_G299V_, or *hly*_C484*_ gene. LLO detection was performed by using LLO-specific antibodies, and InlC-specific antibodies were used as loading controls. (B) qRT-PCR quantification of *hly* transcripts produced in BHI broth at 37°C by the EGDΔ*hly* strain complemented with the pPL2 plasmid alone or containing the *hly*_WT_, *hly*_G299V_, or *hly*_C484*_ gene. Each strain was tested at least three times using independent precultures. *gyrB* was used as a stable reference gene for normalization. Results are shown as fold change of *hly* expression relative to that in EGD (RQ, relative quantities). Each central bar represents the mean of at least three replications. Error bars indicate standard deviations from the means. (C) *In vivo* characterization of the *hly*_G299V_ and *hly*_C484*_ mutant strains compared to the *hly*_WT_ strain. Each BALB/c mouse was infected intravenously with 1 × 10^4^ CFU. Animals were sacrificed 72 h after infection. Numbers of CFU per organ are shown for all tested strains. No bacteria could be recovered from the liver of mice infected with the EGDΔ*hly*::pPL2-*hly*_G299V_ and EGDΔ*hly*::pPL2 strains or from the spleen of mice infected with EGDΔ*hly*::pPL2. Statistical analyses were performed with the Mann-Whitney *U* test, by comparing results with those of EGDΔ*hly*::pPL2-*hly*_WT_. *, *P* < 0.05; **, *P* < 0.01.

Western blot analyses of culture supernatants detected smaller amounts of LLO produced by EGDΔ*hly*::pPL2-*hly*_G299V_ and EGDΔ*hly*::pPL2-*hly*_C484*_ bacteria than by the WT EGD and EGDΔ*hly*::pPL2-*hly*_WT_ strains (Fig. 3A). qRT-PCR analyses showed that the *hly* transcription levels in both the EGDΔ*hly*::pPL2-*hly*_G299V_ and EGDΔ*hly*::pPL2-*hly*_C484*_ strains are comparable to the level observed in EGDΔ*hly*::pPL2-*hly*_WT_ although the level is slightly weaker in EGDΔ*hly*::pPL2-*hly*_C484*_ (Fig. 3B). Furthermore, the EGDΔ*hly*::pPL2-*hly*_C484*_ mutant produced a shorter LLO protein than strains harboring the *hly*_WT_, confirming that the premature stop codon identified in *hly* in the CLIP 1989/13656 strain leads to the production of a truncated LLO. The *hly*_N261*_ mutation (Fig. 1; Table S1) was not tested *in vitro* as this premature stop codon is upstream of the *hly*_C484*_ mutation, leading to an even shorter LLO.

### Virulence of *hly*_G299V_ and *hly*_C484*_ mutants.

We finally assessed the virulence of the EGDΔ*hly*::pPL2-*hly*_G299V_ and EGDΔ*hly*::pPL2-*hly*_C484*_ complemented strains relative to that of the EGDΔ*hly*::pPL2-*hly*_WT_ and EGDΔ*hly*::pPL2 strains upon intravenous injection in mice. The EGDΔ*hly*::pPL2-*hly*_G299V_ and EGDΔ*hly*::pPL2-*hly*_C484*_ strains were four orders of magnitude less abundant than the EGDΔ*hly*::pPL2-*hly*_WT_ strain in the liver and the spleen (Fig. 3C). This demonstrates that the virulence of L. monocytogenes expressing either LLO_G299V_ or LLO_C484*_ is strongly attenuated *in vivo*.

## DISCUSSION

Virulence gene polymorphisms leading to L. monocytogenes attenuation have been previously described and have been associated with strains of lower pathogenic potential. The best characterized are those affecting the invasion-associated *inlA* gene, found in a large proportion (>25 to 30%) of lineage II food isolates but extremely rare among lineage I strains, which are more frequently associated with clinical cases (13, 21, 29, 30). Mutations leading to more radical L. monocytogenes virulence attenuation have also been characterized, particularly those affecting the *prfA* gene (31–33), but their frequency and distribution across the L. monocytogenes population remained undetermined. Here, we examined the occurrence of loss-of-virulence mutations in L. monocytogenes by screening a wide and diverse panel of strains for hemolytic activity. Our data show that nonhemolytic L. monocytogenes mutants occur at low frequency (0.1%) and are phylogenetically diverse, including strains belonging to hypovirulent and hypervirulent clonal complexes (14). This indicates that the underlying mutational events are not linked to the genetic background of the strains.

The L. monocytogenes hemolytic phenotype depends on two essential virulence determinants, the central virulence regulator PrfA and LLO, encoded by *prfA* and *hly*, respectively. Indeed, all nonhemolytic strains identified in this study carried mutations in at least one of these genes. The large majority of nonhemolytic strains (56/60; 93.3%) carried *prfA* mutations (frameshifts, missense or nonsense nucleotide changes, or reversion of the stop codon into a glutamine codon). Although no PrfA activity could be detected and *hly* was not transcribed in these strains, *prfA* was transcribed at levels similar to the level in strain EGDe. This suggests that the loss of PrfA activity in these strains likely results from PrfA misfolding, instability, and/or inactivating amino acid substitution. Some inactivating amino acid substitutions in PrfA occurred in the β-roll, HTH motif, or C-terminal domain, in line with the critical role of these regions in PrfA activity (25–27, 31). As PrfA is the major transcriptional regulator of the virulence genes of L. monocytogenes and is essential for its pathogenicity (23, 34), the virulence of PrfA^−^ strains is expected to be highly attenuated, as previously described (31–33). The first L. monocytogenes strain naturally producing a C-terminally extended PrfA polypeptide (55 residues longer) was identified in this study and showed no PrfA activity and no *hly* transcription.

Nonhemolytic *hly* mutants with affected LLO activity were less frequent (5/60; 8.3%) in our study than strains with loss-of-hemolysis mutations in *prfA*. Our analysis identified for the first time a spontaneous amino acid substitution in LLO (*hly*_G299V_) and premature stop codons in *hly* (*hly*_N261*_ and *hly*_C484*_) leading to the loss of LLO activity. Lower quantities of LLO were detected in the culture supernatants of the EGDΔ*hly*::pPL2-*hly*_G299V_ and EGDΔ*hly*::pPL2-*hly*_C484*_ constructs than in the EGD and EGDΔ*hly*::pPL2-*hly*_WT_ strains. The quantities of *hly* transcripts were similar in the EGDΔ*hly*::pPL2-*hly*_G299V_ and the EGDΔ*hly*::pPL2-*hly*_WT_ control strain, indicating that LLO_G299V_ is likely less stable than WT LLO. In contrast, EGDΔ*hly*::pPL2-*hly*_C484*_ showed a lower *hly* transcription level than that of WT *hly*, suggesting an impaired stability of the *hly*_C484*_ transcript. *In vivo* experiments confirmed that the nonhemolytic strains harboring the *hly*_G299V_ or *hly*_C484*_ mutation have strongly attenuated virulence in mice. In line with these results, only three nonhemolytic strains were isolated from human samples. Although we did not have access to the detailed clinical data of these patients (dating back to the 1980s and 1990s), one possibility would be that they were heavily immunocompromised, mirroring previous reports on isolation of the nonpathogenic L. monocytogenes relative Listeria innocua from immunosuppressed individuals (35).

One of the LLO-negative (LLO^−^) strains (CLIP 1998/76801) underwent huge genomic rearrangements that likely caused the loss of *hly* and *gshF*, encoding a glutathione synthase reported as being required for PrfA activity (28). CLIP 1998/76801 is the only strain in our entire genome database (∼4,100 entries) that lacks *gshF*. Interestingly, each copy of the transposable element that likely caused the genomic rearrangements observed in this strain carried putative cadmium resistance determinants that could be advantageous in environments in which virulence determinants are not needed. Similar transposable elements were detected in monophyletic groups of CC1 and CC59 strains, suggesting that they have been horizontally transmitted in the L. monocytogenes population.

The predominance of PrfA^−^ mutants among the nonhemolytic strains could reflect the fact that *prfA* is a pleiotropic regulatory gene that controls the expression of a number of virulence determinants, the expression of which is known to entail a significant fitness cost under nonhost conditions (24). Our results show that, at 22°C, the majority of PrfA^−^ strains have a fitness level similar to that of EGDe, suggesting that the absence of PrfA activity does not impact L. monocytogenes fitness under nonpathogenic conditions. Nevertheless, reduced fitness was observed at 37°C (mammalian host temperature) compared to that of EGDeΔ*prfA*. This result suggests that nonhemolytic strains are more adapted to a nonpathogenic lifestyle, independently of PrfA. Consistent with this, most of the nonhemolytic L. monocytogenes isolates were from nonclinical origins. The ratio of nonsynonymous to synonymous substitutions (*dN*/*dS*) estimated for *prfA* (*dN*/*dS* = 0.08892) and *hly* (*dN*/*dS* = 0.03674) using a data set of 100 genomes representative of L. monocytogenes phylogenetic diversity (14) confirmed that, similar to L. monocytogenes core genes (*dN*/*dS* = 0.05353, on average [21]), these genes are under purifying selection. Thus, any deleterious mutations affecting these genes tend to be eliminated from the L. monocytogenes population. The relatively low frequency of deleterious mutations in *prfA* and *hly* indicates that there might be a strong selection for L. monocytogenes to retain its virulence capacity (36). Our results also suggest that once strains lose their virulence capacity (e.g., due to a *prfA* mutation), other virulence genes may become irrelevant and prone to accumulate mutations, as observed in our PrfA^−^/LLO^−^ and PrfA^−^/GshF^−^ strains. Previous studies have already identified strains with multiple mutations occurring in several major virulence genes (20). Strains with virulence-attenuating mutations are therefore prone to enter into an evolutionary path toward obligate saprophytism. The L. monocytogenes phylogenomic clade comprises another pathogenic species, Listeria ivanovii, which contains a set of PrfA-regulated genes, as well as nonpathogenic species, some of which contain remnants thereof (e.g., Listeria seeligeri or L. innocua) (37, 38). While infrequent, spontaneous virulence-disabling mutations such as those described here could have been key initial events in the emergence and evolution of the L. monocytogenes-related nonpathogenic Listeria species.

## MATERIALS AND METHODS

### Bacterial strains and growth media.

The 60 nonhemolytic L. monocytogenes isolates included in this study were identified among a collection of 57,820 L. monocytogenes strains collected between 1987 and 2008 by the French National Reference Centre for Listeria (NRCL) and World Health Organization Collaborating Centre for Listeria (WHOCCL) in the context of the epidemiological surveillance of listeriosis. This global collection included isolates of food (*n* = 36,630), clinical (*n* = 5,980), environmental (*n* = 3,647), veterinary (*n* = 1,713), and unknown (*n* = 9,850) origins. Isolates were revived by plating them onto Columbia agar, and single colonies were grown on Columbia agar slants. L. monocytogenes strains were routinely grown in BHI broth at 37°C, and Escherichia coli strains were grown at 37°C in LB broth or agar plates.

### Phenotypic characterization of Listeria isolates.

Miniaturized enzymatic and sugar fermentation tests (API-Listeria identification microgallery; bioMérieux, France), in combination with the hemolytic activity assessment of strains, were used for phenotypic identification of Listeria species (39). Hemolytic activity was tested on Columbia horse blood agar plates (bioMérieux, France). L. monocytogenes CLIP 74910 and Listeria innocua CLIP 74915 were used as positive and negative controls of hemolysis, respectively.

### Genome sequencing and analyses.

Genomic DNA was extracted using a DNeasy Blood and Tissue Extraction kit (Qiagen, Denmark) and used for whole-genome sequencing on an Illumina NextSeq 500 (2 × 150 bp) platform (Illumina, CA, USA). Reads were trimmed with AlienTrimmer (40) to eliminate adapter sequences and discard reads with Phred scores of ≤20. *De novo* assembly of Illumina reads was performed using SPAdes Genome Assembler, version 3.1 (41). The complete genome of the CLIP 1998/76801 strain was obtained by PacBio RS II sequencing (Pacific Biosciences, CA, USA) using DNA purified with a Wizard genomic DNA purification kit (Promega, WI, USA). Genome annotation was performed using Prokka, version 1.11 (42).

PCR serogroups (12, 43), MLST profiles (13), and cgMLST profiles (21) were deduced from genome assemblies using the BIGSdb-L. monocytogenes platform (http://bigsdb.pasteur.fr/listeria) (21). Genome assemblies were made publicly available in the BIGSdb-L. monocytogenes platform (see Table S1 in the supplemental material).

### Assessment of *prfA* and *hly* evolutionary trends.

*prfA* and *hly* sequences were extracted from 100 genomes that were selected to represent the species diversity based on MLST and pulsed-field gel electrophoresis (PFGE) typing (14) and aligned using Muscle, version 3.8 (44). This data set included genomes from 13 food isolates, 45 human clinical isolates, 19 animal isolates, 1 environmental isolate, and 22 isolates of unknown origin. They comprised 41 genomes of lineage I, 53 of lineage II, 5 of lineage III, and 1 of lineage IV and represented 5 singletons and 34 clonal complexes based on MLST. No nonhemolytic strain was included in this analysis. Alignments were used to estimate the ratios of nonsynonymous to synonymous substitutions (*dN*/*dS*) in *prfA* and *hly* using the codeml program included in the PAML package, version 4.4 (45).

### Assessment of PrfA activity.

PrfA activity was assessed by measuring the activity of PrfA-regulated *plcB* and *hpt* gene products as previously described (46, 47). For PlcB, lecithinase tests were performed in egg yolk BHI agar, and for Hpt, glucose-1-phosphate acidification tests were carried out in phenol red broth, in both cases with and without 0.5% (wt/vol) activated charcoal (Merck, NJ, USA). Medium supplementation with charcoal leads to the partial activation of PrfA, presumably due to sequestration of repressor substances from the culture medium. Three L. monocytogenes genotypes from strain P14 were used as controls: (i) a *prfA*_WT_ strain characterized by an activatable PrfA phenotype (lack of PlcB and Hpt activity in normal medium and strong activity in charcoal-supplemented medium), (ii) a Δ*prfA* strain which remains negative for PlcB and Hpt activity in the presence of charcoal, and (iii) a constitutively activated *prfA* (prfA*) strain with strong PlcB and Hpt activity independent of charcoal supplementation (24, 46, 48).

### RNA extractions.

Nonhemolytic strains and EGDΔ*hly*::pPL2-*hly*_WT_, EGDΔ*hly*::pPL2, EGDΔ*hly*::pPL2-*hly*_G299V_, and EGDΔ*hly*::pPL2-*hly*_C484*_ constructs were cultured overnight on BHI agar at 37°C. One colony was used to inoculate 5 ml of BHI broth. After overnight growth at 37°C, 500 μl of culture was added to 10 ml of BHI broth, and the whole exponential-phase culture (at 37°C) was centrifuged at 5,000 × *g* for 5 min. The pellet was suspended with 400 μl of resuspension buffer (10% glucose, 12.5 mM Tris, 10 mM EDTA in nuclease-free water) and transferred to a lysing tube (containing 0.1 mm of ceramic breads, 500 μl of acid phenol, and 60 μl of 0.5 M EDTA). A Precellys 24 homogenizer (Bertin Instruments, France) was used at 6,500 rpm for 23 s two times (10-s break), and the resulting mixture was centrifuged at 14,000 × *g* at 4°C for 10 min. The upper aqueous phase was transferred into a tube containing 1 ml of TRIzol and 100 μl of chloroform, mixed by inversions, and centrifuged. The upper aqueous phase was transferred into a tube containing 200 μl of chloroform, mixed by inversions, and centrifuged. The upper aqueous phase was transferred into a storage tube (containing 650 μl of isopropanol and 65 μl of 3 M sodium acetate), mixed by inversions, precipitated for 20 min at −20°C, and centrifuged for 20 min. The supernatant was rinsed twice with 75% ethanol. The air-dried pellet was dissolved in 300 μl of nuclease-free water. RNA concentrations were measured with a DeNovix DS-11 spectrophotometer (DeNovix, DE, USA) and diluted to obtain 500 ng of RNA in 12.5 μl of nuclease-free water.

### Quantification of *hly* and *prfA* transcripts by qRT-PCR.

For the qRT-PCRs, cDNAs were generated prior to quantitative PCRs (qPCRs). DNase treatment was performed with RNase-free DNase I (New England BioLabs, MA, USA) according to the instructions of the manufacturer. Briefly, 0.5 μl of RNAseOUT, 0.5 μl of DNase I, and 1.5 μl of 10× buffer were added to the 12.5 μl of diluted RNAs, followed by a final addition of 1.5 μl of 0.05 M EDTA. cDNAs were generated by reverse transcription using Moloney murine leukemia virus (MMLV) reverse transcriptase (Invitrogen, CA, USA) and random hexamers for priming according to the instructions of the manufacturer. Briefly, 2 μl of 10 mM deoxynucleoside triphosphates (dNTPs), 2 μl of 2.5 μM random primers, and 3.5 μl of nuclease-free water were added to the 16.5 μl of the previously DNase-treated sample, and then 8 μl of 5× first-strand buffer, 4 μl of 0.1 M dithiothreitol (DTT), and 2 μl of nuclease-free water were added, followed by 0.5 μl of MMLV reverse transcriptase.

All quantitative PCRs were prepared using SYBR green real-time PCR master mixes and a StepOnePlus real-time PCR system (Applied Biosystems, CA, USA). Each primer pair was used for separate reactions using PCR mixtures containing 1 μl of a 9 μM concentration of each primer (Table S2), 5 μl of SYBR mix, 1 μl of cDNA diluted at 1:5, and 3 μl of nuclease-free water. Real-time PCRs were carried out in MicroAmp Fast Optical 96-well reaction plates (Applied Biosystems, CA, USA) using the following protocol: initial denaturation at 95°C for 10 min, followed by 40 cycles of denaturation at 95°C for 15 s and primer annealing/elongation at 60°C for 1 min. Each strain was tested at least three times using independent precultures. *gyrB* was used as a stable reference gene for normalization. Results are shown as fold change of the target gene expression level relative to that of EGDe or EGD (relative quantities [RQ]), which was deduced from the cycle threshold (*C_T_*) values using the 2^−ΔΔ*CT*^ methodology.

### Fitness studies.

The microbial growth of nonhemolytic strains, EGDe, EGD, and EGDeΔ*prfA* was monitored over time in BHI broth at 22°C and 37°C using absorbance measurements (optical density at 600 nm [OD_600_]) through a Bioscreen C system (Oy Growth Curves Ab Ltd., Helsinki, Finland). Bacteria were first cultured overnight on BHI agar at 22°C or 37°C, and one colony was used to inoculate 5 ml of BHI broth. After overnight growth, the stationary-phase cultures were diluted to reach an OD_600_ of 0.1 and transferred into Bioscreen C 96-well plates. The OD_600_ values of noninoculated wells (blanks) were subtracted from those of inoculated ones to delete the background noise. Each strain was tested three times. Mean OD_600_ values per strain were used to calculate the areas under the curves over time. For this, data were fitted to parametric models (Gompertz, modified Gompertz, logistic, and Richards laws) using the gcFit function of the grofit R package, version 1.1.1-1 (49). The model that best fitted the data was selected by means of an Akaike information criterion (AIC) (50) and used to derive areas under the growth curves.

### DNA manipulations and cloning.

We used a two-step cloning strategy to introduce the wild-type *hly* (*hly*_WT_), *hly*_G299V_, or *hly*_C484*_ genes in the L. monocytogenes strain EGDΔ*hly*. First, we cloned separately the *hly*_WT_, *hly*_G299V_, and *hly*_C484*_ gene sequences into the Listeria integrative vector pPL2 (51). Primers used are listed in Table S2. To deliver plasmids into L. monocytogenes, Escherichia coli S17.1 (colistin and nalidixic acid sensitive) was transformed with the plasmids, followed by conjugation with L. monocytogenes EGDΔ*hly* (colistin and nalidixic acid resistant). L. monocytogenes EGDΔ*hly* bacteria were selected on 7 μg/ml chloramphenicol (bacteria containing the pPL2 derivatives), 10 μg/ml colicin, and 50 μg/ml nalidixic acid (selection of resistant L. monocytogenes versus sensitive E. coli bacteria). Since all our constructs were made on a similar EGD background, the PrfA* phenotype of EGD was not expected to have any impact on our results and conclusions.

### Western blotting.

Protein extracts were obtained from EGD, EGDΔ*hly*, EGDΔ*hly*::pPL2, EGDΔ*hly*::pPL2-*hly*_WT_, EGDΔ*hly*::pPL2-*hly*_G299V_, and EGDΔ*hly*::pPL2-*hly*_C484*_ as follows. Bacteria were grown overnight in BHI broth at 37°C. After centrifugation of bacterial cultures (30 min at 2,151 × *g*), all proteins of the supernatant were precipitated by using trichloroacetic acid (20%) and washed using acetone. Proteins were then separated by SDS-PAGE (8% acrylamide gel and 3.9% stacking gel) and transferred to a polyvinylidene difluoride transfer membrane (Bio-Rad, CA, USA). The membrane was incubated overnight at 4°C with a blocking buffer containing dried milk (5%), phosphate-buffered saline (PBS; 1%), and Tween (0.1%) and washed with PBS (1%) and Tween (0.1%). The membrane was then incubated first with a polyclonal anti-LLO (52, 53) or anti-InlC antibody (54) (1/20,000; 1 h at room temperature) and second with an anti-rabbit antibody (1/3,000; 1 h at room temperature). The membrane was washed with PBS (1%) and Tween (0.1%) between each incubation step with antibodies. Antibody-antigen interactions were revealed using a SuperSignal West Pico chemiluminescent substrate (Thermo Fischer Scientific, MA, USA).

### Animal studies.

The virulence of L. monocytogenes strains EGDΔ*hly*::pPL2-*hly*_WT_, EGDΔ*hly*::pPL2-*hly*_G299V_, EGDΔ*hly*::pPL2-*hly*_C484*_, and EGDΔ*hly*::pPL2 was assessed *in vivo*. BALB/c mice were infected via the intravenous route with 1 × 10^4^ CFU per animal. At 72 h postinfection, mice were sacrificed for spleen and liver dissection. CFU were enumerated by plating dilutions of the whole homogenized organs onto BHI plates. Statistical analyses were performed with the Mann-Whitney *U* test, by comparing the results with those of EGDΔ*hly*::pPL2-*hly*_WT_. All procedures were in agreement with the guidelines of the European Commission for the handling of laboratory animals, directive 86/609/EEC, and were approved by the Animal Care and Use Committee of the Institut Pasteur, as well as by the ethical committee of Paris Centre et Sud under the number 2010-0020.

## Supplementary Material

Supplemental material
