# Supplementary material for: Spontaneous Loss of Virulence in Natural Populations of Listeria monocytogenes
Source: Infect Immun. 2017 Oct 18;85(11):e00541-17. doi: 10.1128/IAI.00541-17 (PMC5649026; doi:10.1128/IAI.00541-17)
Supplement: Supplemental material [file supp_85_11_e00541-17__index.html]

Supplemental material 

# Spontaneous Loss of Virulence in Natural Populations of Listeria monocytogenes

## Supplemental material

- Supplemental file 1 -

  Table S1. Characteristics and amino acid modifications identified in PrfA and LLO for the 60 nonhemolytic strains.

  XLSX, 31K
- Supplemental file 2 -

  Table S2. Primers used in this study.

  XLSX, 12K
- Supplemental file 3 -

  Fig. S1. Fitness analyses of the nonhemolytic strains.

  PDF, 127K
- Supplemental file 4 -

  Fig. S2. Assessment of PrfA activity.

  PDF, 190K
- Supplemental file 5 -

  Fig. S3. Quantification of *prfA* and *hly* transcripts for a representative set of nonhemolytic strains.

  PDF, 201K
- Supplemental file 6 -

  Legends for Fig. S1 to S3 and Tables S1 and S2.

  PDF, 88K
