# Supplementary figures and images for: Spontaneous Loss of Virulence in Natural Populations of Listeria monocytogenes
Source: Infect Immun. 2017 Oct 18;85(11):e00541-17. doi: 10.1128/IAI.00541-17 (PMC5649026; doi:10.1128/IAI.00541-17)

Figure S1

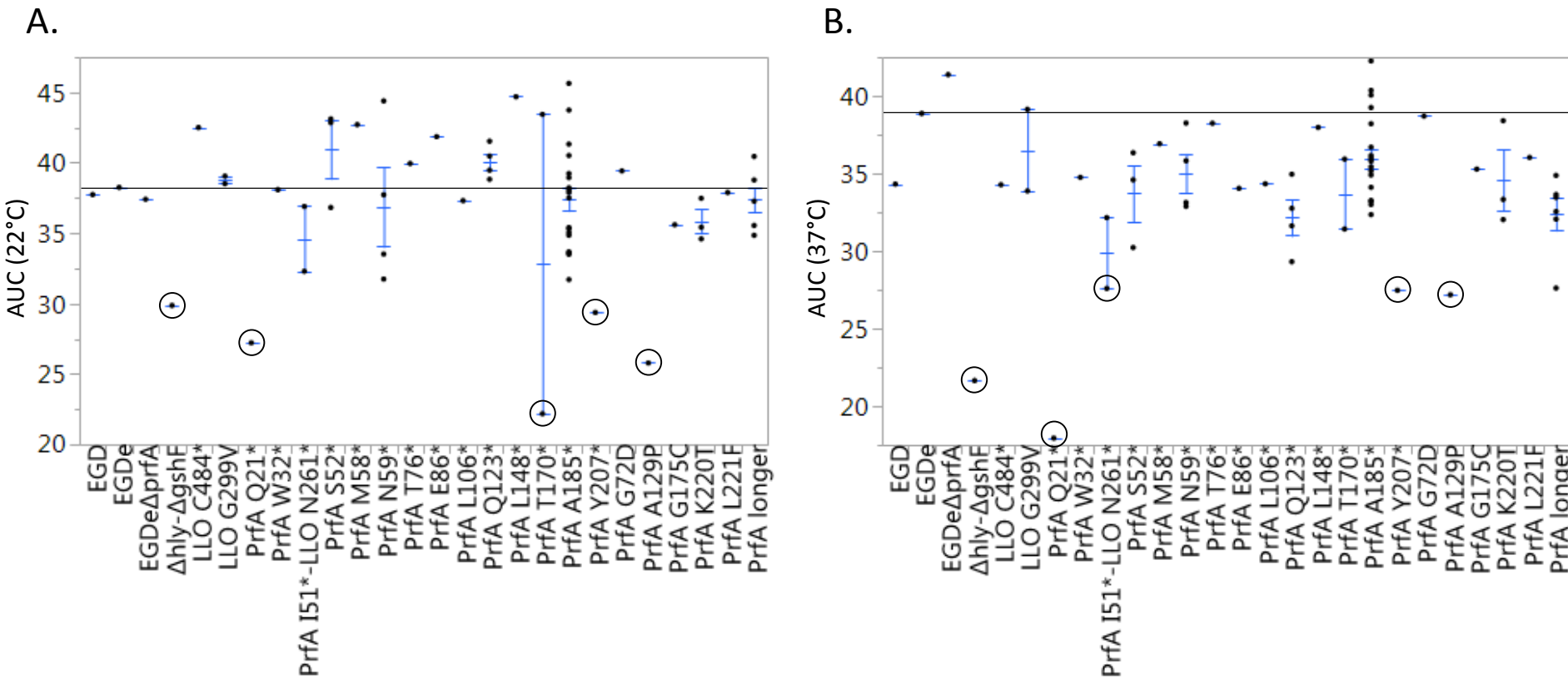

Supplement: Supplemental material [file IAI.00541-17_zii999092197s3.pdf]

Figure S2

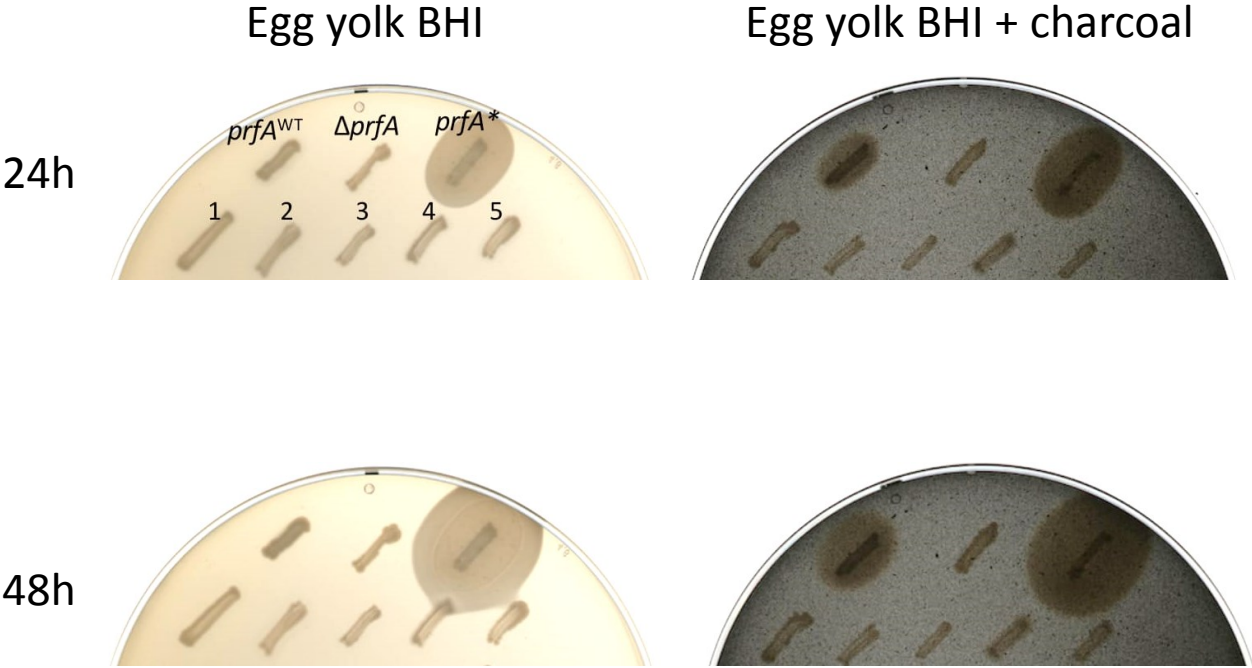

Supplement: Supplemental material [file IAI.00541-17_zii999092197s4.pdf]

Figure S3

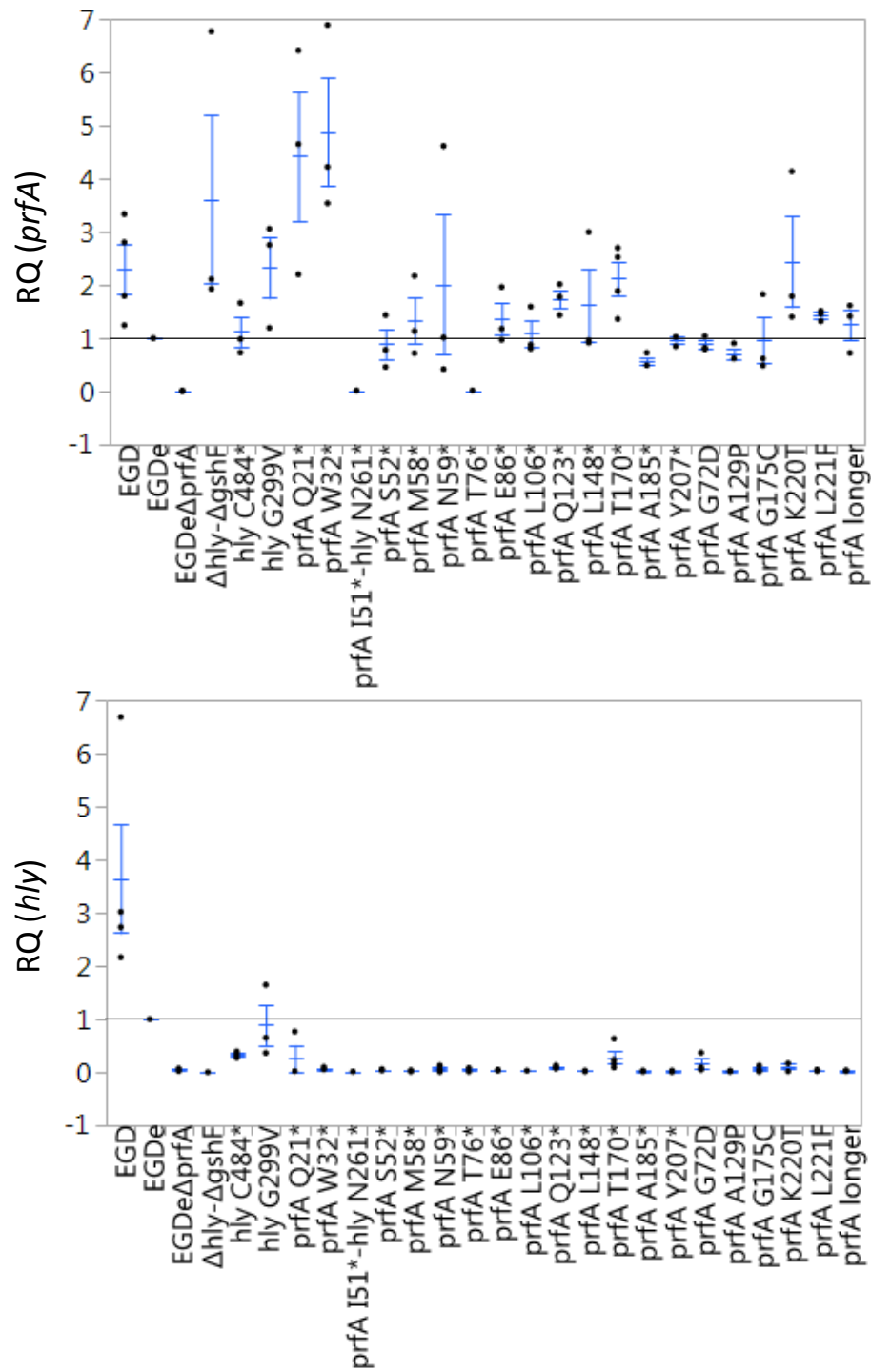

Supplement: Supplemental material [file IAI.00541-17_zii999092197s5.pdf]
