# Supplementary material for: Spontaneous Loss of Virulence in Natural Populations of Listeria monocytogenes
Source: Infect Immun. 2017 Oct 18;85(11):e00541-17. doi: 10.1128/IAI.00541-17 (PMC5649026; doi:10.1128/IAI.00541-17)
Supplement: Supplemental material [file IAI.00541-17_zii999092197s6.pdf]

Supplementary figure 1: Fitness analyzes of the non-hemolytic strains.

Growth analyzes of all non-hemolytic strains were performed in BHI at 22°C (A) and 37°C (B). Areas under the growth curves (AUC) were calculated for each strain using the "gcFit" function of the "grofit" R package v.1.1.1-1. AUC values are shown for each strain (being represented by one black dot) per type of loss-of-hemolysis mutation. Each central bar represents the mean of at least three replications. Error bars indicate standard deviations from the means. Horizontal black lines indicate the AUC values obtained for EGDe. Black circles surround strains that show the most impaired growth.

Supplementary figure 2: Assessment of PrfA activity.

PrfA activity was assessed using, as reporter, the lecithinase activity of PlcB on egg yolk BHI agar plates both in PrfA-non-activating (without charcoal, left) and -activating conditions (with 0.5% w/v activated charcoal, right). Two time points are shown: 24 h (above) and 48 h (below). $prfA_{WT}$: $P14_{WT}$; Δ*prfA*: P14Δ*prfA*; *prfA**: P14*prfA**; 1: CLIP 2008/01432; 2: CLIP 1996/70860; 3: CLIP 2001/89406; 4: CLIP 2008/01435; 5: CLIP 2001/89407. Positions of the strains are identical in all pictures. All non-hemolytic strains showed similar activity profiles as strains 1 to 5.

Supplementary figure 3: Quantification of *prfA* and *hly* transcripts for a representative set of non-hemolytic strains.

Fold change of *prfA* (above) and *hly* (below) transcription relative to EGDe (Relative quantities, RQs) are shown for a representative set of non-hemolytic strains (**Table S1**, one strain per type of loss-of-hemolysis mutation). *prfA* and *hly* expression levels were normalized according to *gyrB* expression. Each central bar represents the mean of at least

three replications. Error bars indicate standard deviations from the means. Horizontal black lines indicate the RQs of 1 on the y-axes.

Supplementary Table 1: Characteristics and amino-acid modifications identified in PrfA and LLO for the 60 non-hemolytic strains.

Lineages, PCR serogroups, sequence types and clonal complexes (MLST) as well as cgMLST types were deduced from genome sequences using the BIGSdb-*Lm* platform (http://bigsdb.pasteur.fr/listeria). All genome assemblies are publicly available in the BIGSdb-*Lm* platform using the identifiers listed in the first column. Assemblies of both Illumina and PacBio reads are made available for the CLIP 1998/76801 strain. Positions of amino-acid substitutions and stop codons in PrfA and LLO are indicated. Stop codons are indicated with black stars and highlighted in blue; amino acid substitutions are highlighted in orange. No *bglA* allele could be deduced based on the Illumina reads of the CLIP 2004/96369 strain, and no ST could therefore be assigned, but a clonal complex could be deduced based on the 6 other MLST alleles. Strains used for the quantification of *hly* and *prfA* transcripts are notified in the "Used in *hly* and *prfA* qRT-PCR assays" column.

Supplementary table 2: Primers used in this study.

All primers used for *hly* complementation in a EGDΔ*hly* background (EGDΔ*hly*:pPL2-hly_{WT}, EGDΔ*hly*:pPL2-hly_{G299V} and EGDΔ*hly*:pPL2-hly_{C484*} constructs) as well as the primers used for qPCR assays are listed. Restriction enzymes recognition sites are underlined.
